# Preclinical insights into gamma-tACS: foundations for clinical translation in neurodegenerative diseases

**DOI:** 10.3389/fnins.2025.1549230

**Published:** 2025-03-12

**Authors:** Guillermo Sánchez-Garrido Campos, Ángela M. Zafra, Marta Estévez-Rodríguez, Isabel Cordones, Giulio Ruffini, Javier Márquez-Ruiz

**Affiliations:** ^1^Department of Physiology, Anatomy and Cell Biology, Pablo de Olavide University, Seville, Spain; ^2^Brain Modeling Department, Neuroelectrics Barcelona, Barcelona, Spain

**Keywords:** non-invasive brain stimulation (NIBS), gamma-tACS, transcranial electric stimulation, Parkinson’s disease, Alzheimer disease, animal model

## Abstract

Gamma transcranial alternating current stimulation (gamma-tACS) represents a novel neuromodulation technique with promising therapeutic applications across neurodegenerative diseases. This mini-review consolidates recent preclinical and clinical findings, examining the mechanisms by which gamma-tACS influences neural oscillations, enhances synaptic plasticity, and modulates neuroimmune responses. Preclinical studies have demonstrated the capacity of gamma-tACS to synchronize neuronal firing, support long-term neuroplasticity, and reduce markers of neuroinflammation, suggesting its potential to counteract neurodegenerative processes. Early clinical studies indicate that gamma-tACS may improve cognitive functions and network connectivity, underscoring its ability to restore disrupted oscillatory patterns central to cognitive performance. Given the intricate and multifactorial nature of gamma oscillations, the development of tailored, optimized tACS protocols informed by extensive animal research is crucial. Overall, gamma-tACS presents a promising avenue for advancing treatments that support cognitive resilience in a range of neurodegenerative conditions.

## Introduction

Transcranial electrical stimulation (tES) is a non-invasive technique that modulates brain activity by applying low-intensity electrical currents to the scalp through strategically placed electrodes, positioned according to the specific brain area targeted in each application. Low-intensity stimulation, typically ranging from 1 to 2 mA, is chosen for its safety and effectiveness in modulating cortical excitability without discomfort or adverse effects, while staying below the threshold for direct neuronal firing. These currents can alter cortical excitability with both immediate and lasting effects (Nitsche and Paulus, 2000; Woods et al., 2016). The primary forms of tES are transcranial direct current stimulation (tDCS), transcranial alternating current stimulation (tACS), and transcranial random noise stimulation (tRNS). tACS uses oscillating currents to synchronize neuronal activity at specific frequencies, aligning with brain rhythms associated with cognitive and behavioral functions (Herrmann et al., 2013). This frequency-specific entrainment allows tACS to target conditions such as schizophrenia, epilepsy, and Alzheimer’s disease (AD) by modulating neural connectivity (Antal and Paulus, 2013; Kasten et al., 2016). Gamma oscillations span a wide range of frequencies (30–80 Hz), with evidence showing that slow-gamma (usually slower than 40 Hz) are primarily involved in memory consolidation and synaptic plasticity, and fast-gamma (usually faster than 40 Hz) more closely associated with attentional processes and perceptual binding, facilitating communication across brain networks (Buzsáki and Wang, 2012; Fries, 2015; Murty et al., 2020). Gamma-band activity has been linked to key cognitive domains, including working memory (Tallon-Baudry et al., 1999), sensory processing (Gray and Singer, 1989), and motor control (Cheyne and Ferrari, 2013). These oscillations are fundamental to cognitive flexibility and neural computation, as they facilitate long-range communication between distant cortical and subcortical structures (Roux and Uhlhaas, 2014). Dysregulation of gamma rhythms is associated with cognitive deficits in neurodegenerative diseases like AD, where reduced gamma power and coherence are linked to pathological markers, including amyloid-beta (Aβ) and tau aggregation (Stam, 2010; Uhlhaas and Singer, 2010). In Parkinson’s disease (PD), while beta-band abnormalities have been more extensively studied, gamma-band activity has also been implicated in motor functions, with studies hypothesizing that reduced gamma oscillations may correlate with bradykinesia and motor rigidity (Muthuraman et al., 2021), and suggesting that they could contribute to movement initiation and execution (Lofredi et al., 2018). Given this background, gamma-tACS has shown promise in enhancing neural synchrony, synaptic plasticity, and microglial responses, with animal studies demonstrating neuroprotective effects and reduced Aβ accumulation (Martorell et al., 2019; Adaikkan and Tsai, 2020). Clinical trials further report improvements in cognitive functions, evidenced by increased hippocampal perfusion and memory gains in AD patients (Sprugnoli et al., 2021; Dhaynaut et al., 2022). These findings underscore the therapeutic potential of gamma-tACS for addressing oscillatory dysfunctions in neurodegenerative and psychiatric disorders (Herrmann et al., 2016; Polanía et al., 2018). This mini-review synthesizes preclinical and clinical evidence on the therapeutic potential of gamma-tACS for neurodegenerative diseases, focusing on AD and PD. Key gaps in understanding gamma-tACS mechanisms are highlighted, together with insights from animal studies informing clinical applications and providing a framework to advance gamma-tACS research to enhance cognitive outcomes.

## Physiological mechanisms of gamma-tACS

The therapeutic effects of tACS rely on its capacity to synchronize neural activity and modulate network dynamics. Understanding its impact on neuronal entrainment, neuroplasticity, and glial interactions is crucial for exploring its clinical potential in neurodegenerative diseases. The following sections will examine these mechanisms, detailing how tACS influences neural oscillations, specific neuronal and glial responses, and promotes lasting neuroplasticity.

### Mechanistic insights into neuronal entrainment by tACS

**Figure 1:**
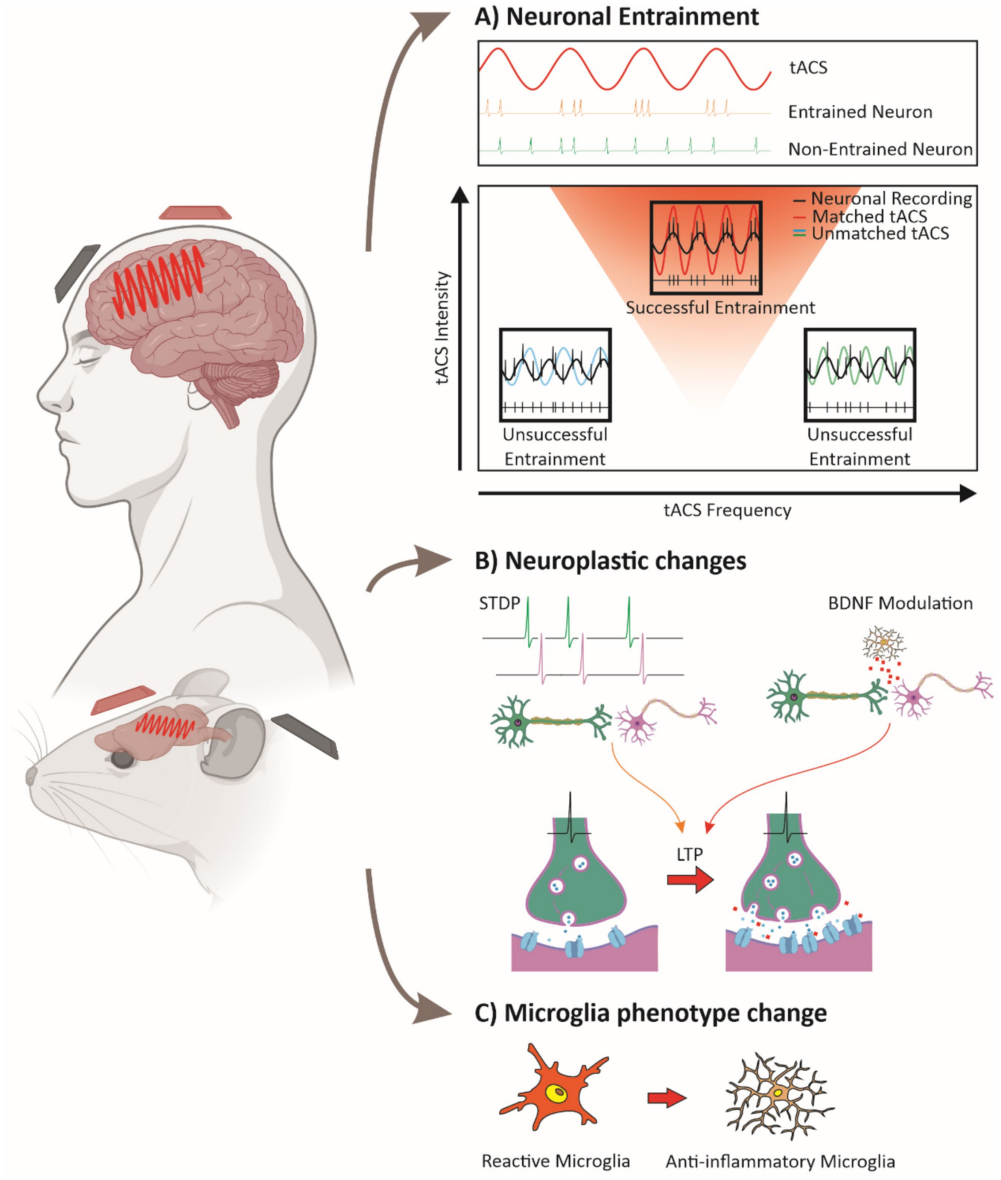
Mechanisms of tACS-induced neural modulation. **(A)** Neuronal entrainment: a sine wave representing tACS is shown alongside neuronal firing patterns. Neurons can synchronize their action potentials to the tACS signal (entrainment, upper panel) or remain unsynchronized (lower panel). The relationship between tACS intensity and frequency is depicted, with a triangular region indicating the frequencies and intensities that promote neuronal entrainment (an Arnold tongue). Warmer colors within the triangle represent stronger entrainment. Superimposed sine waves of tACS at three different frequencies illustrate how neuronal firing synchronizes only within the entrainment region (red triangle). **(B)** Plasticity mechanisms: tACS influences synaptic plasticity through spike-timing-dependent plasticity (STDP) and increased BDNF release from microglia, leading to long-term potentiation (LTP). Enhanced synaptic strength is represented by greater neurotransmitter release and increased receptor density at the postsynaptic membrane. **(C)** Microglial modulation: tACS promotes a transition from pro-inflammatory, amoeboid microglia to an anti-inflammatory, ramified state, highlighting its potential effects on neuroinflammation. Together, these mechanisms illustrate how tACS drives neural synchronization, enhances plasticity, and modulates glial activity to support therapeutic outcomes. (Some images were created and obtained from BioRender website: https://www.biorender.com/).

Neuronal entrainment, or synchronized neuronal firing, is a primary effect of tACS, which uses sub-threshold electric fields to modulate membrane potentials and timing of neuronal firing without directly inducing action potentials (Herrmann et al., 2013, 2016; Romei et al., 2016; Vosskuhl et al., 2016). Preclinical studies in animal models demonstrate that oscillatory cycles of depolarization and hyperpolarization during tACS phase-lock neuronal firing to the stimulation, with the strongest effect occurring when the tACS frequency matches the natural frequency of the circuit, a resonance phenomenon that enhances entrainment (Figure 1A) (Chan et al., 1988; Deans et al., 2007; Radman et al., 2007; Fröhlich and McCormick, 2010; Ozen et al., 2010; Reato et al., 2010; Herrmann et al., 2013, 2016; Krause et al., 2019, 2022; Zhao et al., 2024). This modulation affects firing patterns and network organization, potentially supporting long-term effects on circuits (Radman et al., 2007, 2009; Reato et al., 2010; Helfrich et al., 2014), enabling tACS to restore regular oscillatory patterns in dysfunctional networks (Antal and Paulus, 2013; Herrmann et al., 2016; Zhao et al., 2024). Computational models have further explored these mechanisms, showing how tACS effects depend on intensity and network resonance properties (Zhao et al., 2024). Frequency and intensity parameters are crucial for effective tACS entrainment. Frequencies close to intrinsic oscillations of neural groups produce robust entrainment, a principle often modeled by “Arnold tongues” (Arnol, 1963), where increased intensity widens the range of entrainable frequencies (Figure 1A) (Helfrich et al., 2014; Herrmann et al., 2016; Krause et al., 2022; Zhao et al., 2024). Lower intensities align with neurons at matched frequencies, enhancing synchrony in narrow subsets (Schmidt et al., 2014; Krause et al., 2019), while higher intensities expand the affected range, enabling “reentrainment” of neurons that otherwise do not match the tACS frequency (Schmidt et al., 2014; Vöröslakos et al., 2018; Asan et al., 2020), though stability may decrease at high intensities, potentially disrupting coherence (Moliadze et al., 2012; Schmidt et al., 2014). Balancing frequency and intensity parameters could optimize tACS synchronization effects while preserving network stability.

### tACS and neuroplasticity

A key factor in applying tACS clinically is determining if its effects outlast stimulation, enabling lasting network changes and synaptic plasticity. While immediate phase-locking effects are known, studies suggest tACS may induce enduring synaptic strength changes through spike-timing-dependent plasticity (STDP), which modulates connectivity via timing of pre- and post-synaptic spikes, resulting in long-term potentiation (LTP) or depression (LTD) (Zaehle et al., 2010; Vossen et al., 2015; Wischnewski et al., 2019; Vogeti et al., 2022; Nissim et al., 2023).

Different tACS frequencies have been linked to distinct plasticity-related effects. For instance, Zaehle et al. (2010) found that alpha-tACS increased alpha power, a result they attributed to phase-locking of neural activity, which could potentially involve mechanisms such as spike-timing-dependent plasticity (STDP). This interpretation is supported by subsequent studies suggesting that tACS effects on neural oscillations and plasticity may rely on STDP-related processes, as suggested by Vossen et al. (2015) through computational modeling, and by Wischnewski et al. (2019), who showed that NMDA receptor antagonists can block tACS-induced plasticity. While STDP may play a role, the precise contributions of this mechanism remain under investigation (Herrmann et al., 2016). Beta-tACS may enhance neuroplasticity by upregulating brain-derived neurotrophic factor (BDNF), which supports synaptic stability and potentiation, relevant for memory (Lu and Figurov, 1997; Kojima and Mizui, 2017; Wischnewski et al., 2019; Riddle et al., 2020). BDNF exerts its effects primarily through activation of the tropomyosin receptor kinase B (TrkB), which triggers intracellular signaling cascades that enhance synaptic plasticity and long-term potentiation (LTP) (Lu et al., 2014). Given the role of TrkB in mediating activity-dependent synaptic strengthening, its involvement may be a crucial component of the plasticity effects observed with tACS, though further research is needed to establish this link. Gamma-tACS has been increasingly associated with excitatory plasticity, particularly LTP-like mechanisms. Guerra et al. (2018) demonstrated in humans that combining gamma-tACS with intermittent TMS-induced theta burst stimulation (iTBS) enhanced and prolonged LTP-like plasticity effects in the motor cortex, suggesting that gamma oscillations facilitate synaptic potentiation. Complementarily, Guerra et al. (2019) showed that gamma-tACS can reverse LTD-like plasticity in the primary motor cortex, indicating that gamma oscillations may play a broader regulatory role in synaptic plasticity by counteracting synaptic depression. These findings suggest that gamma-tACS may bias plasticity towards LTP by both enhancing potentiation and mitigating LTD, though the precise conditions under which these effects emerge require further study. Gamma-tACS has also been linked to neurotransmitter modulation, particularly dopamine and glutamate, which are essential for synaptic efficacy in memory-related networks (Wischnewski et al., 2019; Jeong et al., 2021). In AD models, gamma-tACS improved neuronal synchrony and reduced amyloid burden, possibly by upregulating these neurotransmitters in key memory circuits (Jeong et al., 2021). Despite these promising findings, the long-term effects of gamma-tACS remain variable. In healthy subjects, Kasten et al. (2016) found that alpha-tACS produced stronger network synchrony aftereffects, while gamma-tACS showed more variability, indicating that higher frequencies may yield less consistent plasticity (Wischnewski and Schutter, 2017). This aligns with the larger spatial coherence of alpha rhythms compared to gamma, which are more localized and dependent on specific stimuli, making alpha rhythms better suited to the low spatial resolution of tACS (Pascual-Marqui et al., 1995; Buffalo et al., 2010; Jia et al., 2011). This variability underscores the fact that while tACS can facilitate neuroplasticity, the outcomes are influenced by a range of frequencies and individual differences, highlighting the need for further research to establish optimal parameters. This variability is evident not only in gamma frequency stimulation (Tseng et al., 2016; Wessel et al., 2023) but also across other frequencies (Tseng et al., 2018), affecting both healthy subjects and patient populations (Wang et al., 2024). In summary, tACS influences neuroplasticity through STDP, BDNF modulation, and neurotransmitter release (Figure 1B). While immediate synchrony effects are well-documented, sustained plasticity depends on frequency, intensity, and individual neurophysiological factors, making parameter optimization essential for therapeutic use.

### tACS entrainment across neuronal subtypes: morphology and network dynamics

Following the exploration of how tACS entrains neural networks, it is important to consider how neuronal subtypes may respond differently to tACS. Initial theories suggested that tACS may selectively target specific neurons based on their morphology. Pyramidal cells, with elongated morphology and alignment within cortical layers, were thought to be more susceptible to tACS-induced electric fields, while interneurons, with their compact morphology, were believed to be less affected (Radman et al., 2009; Ruffini et al., 2013, 2014; Aberra et al., 2018; Galan-Gadea et al., 2023; Mercadal et al., 2023). However, recent findings challenge this morphology-based assumption. While morphology is likely to play a role at the single cell level, studies indicate that neuronal response to tACS is shaped primarily by network dynamics. In animal models, ‘network dynamics’ often refers to local microcircuits, where interneurons and pyramidal cells interact via inhibitory and excitatory connections at a mesoscale level (Huang et al., 2021; Krause et al., 2022). Additionally, computational models have been used to explore how network interactions shape neuronal responses to tACS, providing insights into the mechanisms underlying oscillatory entrainment (Clusella et al., 2023). Network interactions may amplify tACS effects on interneurons, which can subsequently influence pyramidal cells. In contrast, human studies generally assess network effects in terms of large-scale functional connectivity, measured through electroencephalography (EEG), magnetoencephalography (MEG), or functional magnetic resonance imaging (fMRI), reflecting oscillatory coupling across distant brain regions (Zhao et al., 2024). This network-centered perspective suggests that functional roles within the network, rather than individual cellular morphology alone, are critical in determining tACS effects. This shift toward a network-centric view emphasizes that understanding tACS-induced modulation requires bridging findings from local circuit interactions in animal models to large-scale connectivity patterns in humans, highlighting the complexity of tACS-induced modulation within neural circuits.

### Gamma tACS and glial activity

Recent research highlights the unique, frequency-specific effects of gamma-tACS on neuronal and glial activity, showing promise for modulating neuroinflammatory processes. Studies indicate that gamma-tACS can reduce pathological markers in neurodegenerative diseases by influencing glial responses. Dhaynaut et al. (2022) observed reduced tau accumulation and lower microglial activation in AD patients receiving gamma-tACS, suggesting anti-inflammatory benefits. Similarly, Wu et al. (2022) reported that prolonged gamma tACS in animal models decreased Aβ levels in the hippocampus, associated with shifts in microglial morphology toward a less reactive state (Figure 1C), increased gamma oscillatory power, and spatial memory improvements. However, while reductions in Aβ and tau have been reported following gamma-tACS (Wu et al., 2022; Dhaynaut et al., 2022), these effects do not necessarily equate to decreased neuroinflammation. Rather, the relationship between pathology clearance and neuroinflammation is complex, as microglial activation states can be both a consequence and a modulator of disease pathology (Lull and Block, 2010; Hansen et al., 2018). These findings position gamma-tACS as a promising approach for neuroinflammation, through an impact on neuronal and glial pathways. Exploring these complementary effects may enhance gamma-tACS’s therapeutic efficacy.

## Therapeutic potential of tACS in neurodegenerative diseases: evidence from animal models to clinical applications

Research on neuroplasticity demonstrates tACS’s ability to induce lasting changes in synaptic strength and network connectivity (Zaehle et al., 2010; Kasten et al., 2016; Wischnewski and Schutter, 2017; Guerra et al., 2019; Wischnewski et al., 2019; Riddle et al., 2020; Giustiniani et al., 2021; Jeong et al., 2021). Studies in animal models and clinical populations show that specific frequencies can modulate neuropathology in neurodegenerative diseases like AD and PD (see Table 1). Animal models provide controlled settings to examine the impact of tACS on neuroinflammatory and neuroplastic processes, offering insights into how targeted frequencies influence disease markers. Clinical studies build on these findings, demonstrating frequency-specific therapeutic effects that address motor and cognitive symptoms. These studies form a translational framework for using frequency-specific tACS to target neuropathological mechanisms behind motor and cognitive impairments in neurodegenerative diseases.

**Table 1:** Summary of clinical and preclinical studies investigating the effects of tACS on AD and PD.

Alzheimer’s disease	Publication	Patient/Animal model	Stimulation parameters	Targeted area	Results
Preclinical studies	Jeong et al. (2021)	AD (5XFAD mice model)	40 Hz tACS 200 μA Current density: Not Reported 20 min/2 weeks	Bilateral frontal lobe	Enhancement of synaptic plasticity (field excitatory postsynaptic potential) Increased LTP in the hippocampal CA1 region
	Wu et al. (2022)	AD (APP/PS1 mice model)	40 Hz tACS 130 μA Current density: Not Reported 20 min/7, 14, 21 and 28 days	Right hippocampus	Cognitive improvement Increase in spontaneous gamma Power and gamma-theta cross-frequency coupling between the right hippocampus and the prefrontal cortex Microglial activation and reduction of hippocampal Aβ plaques
	Wu et al. (2023)	AD (APP/PS1 mice model)	40 Hz tACS 135 μA Current density: Not Reported 20 min/21 days	Right hippocampus	Cognitive improvement Increase in spontaneous gamma Power and gamma-theta cross-frequency coupling between the right hippocampus and the prefrontal cortex
Clinical studies	Naro et al. (2016)	MCI-AD patients	Gamma tACS 1 mA Current density: 0.04 mA/cm^2^ 10 min	Multiple cortical areas, including motor and prefrontal cortices	MCI patients showed increased gamma band oscillations and neurophysiological improvement after gamma tACS AD patients showed no significant changes MCI individuals lacking gamma band enhancement after tACS were more likely to progress to AD within 2 years, suggesting potential diagnostic and prognostic value
	Sprugnoli et al. (2021)	AD patients	40 Hz tACS 4 mA Current density: 0.64 mA/cm^2^ 1 h/2–4 weeks	Bilateral temporal lobes, focused on hippocampal regions	Increased cerebral blood flow in bilateral temporal lobes Improvement in episodic memory Increase in gamma band power
	Benussi et al. (2021)	MCI-AD patients	40 Hz tACS 3 mA Current density: 0.09 mA/cm^2^ 60 min	Medial parietal cortex, aimed to focus the precuneus	Improvement in episodic memory Enhanced cholinergic transmission
	Dhaynaut et al. (2022)	AD patients	40 Hz tACS 2 mA Current density: Not reported 1 h/4 weeks	Bilateral temporal lobes	Trend for an increase in gamma oscillations after tACS Decrease of p-Tau burden in temporal lobe regions in 3/4 patients Significant decrease in microglial activation in 1/4 patients No changes regarding Aβ burden or cognitive outcomes
	Cappon et al. (2023)	AD patients	40 Hz tACS < 2 mA Current density: 0.64 mA/cm^2^ 20 min/5 days	Left angular gyrus	Improvement in episodic memory Decreased theta/gamma power ratio in the left angular gyrus

Parkinson’s disease	Publication	Patient/Animal model	Stimulation parameters	Targeted Area	Results
Preclinical studies	Lee et al. (2022)	PD (MPTP-induced mice model)	20 Hz tACS Current density: 8.91 mA/cm^2^ 20 min/5 days	Primary motor cortex	Improvement of motor performance Neuroprotection of dopaminergic neurons in the substantia nigra Increased GDNF production in striatal PV+ interneurons
Clinical studies	Del Felice et al. (2019)	PD patients	4 Hz tACS and 30 Hz tACS 1–2 mA Current density: 0.02–0.06 mA/cm^2^ 30 min/2 weeks	Scalp area with pathological oscillation + ipsilateral mastoid	Theta-tACS (4 Hz) reduced beta-band excess and improved bradykinesia Beta-tACS (30 Hz) showed no significant effects Cognitive function improved at follow-up, suggesting long-term plasticity No significant improvement in gait/posture
	Guerra et al. (2020)	PD patients	70 Hz gamma tACS + iTBS 1.5 mA Current density: 0.04 mA/cm^2^ 20 min	Primary motor cortex	Restoration of LTP-like plasticity Enhanced GABA-A-ergic function Beta tACS improved movement speed
					Gamma tACS enhanced movement amplitude
	Guerra et al. (2022)	PD patients	20 Hz and 70 Hz tACS 1.5 mA Current density: 0.04 mA/cm^2^ 20 min	Primary motor cortex	
	Guerra et al. (2023a)	PD patients	70 Hz tACS + iTBS 1.5 mA Current density: 0.04 mA/cm^2^ 20 min	Primary motor cortex	Enhancement of MEPs Improvement in GABA-A-ergic function by increased SICI Similar effects regardless of L-dopa states
	Guerra et al. (2023b)	PD patients with bradykinesia symptoms	20 Hz tACS 1.5 mA Current density: 0.04 mA/cm^2^ 20 min	Primary motor cortex	Improvement of STP Reduction of bradykinesia symptoms, particularly improvement in movement speed and amplitude

### Animal models of AD and preclinical insights

Animal models are essential in AD research for replicating key pathological features, like amyloidosis, tauopathy, neuroinflammation, and synaptic loss, though they do not fully capture the complexity of human AD due to lifespan and physiological differences (Palop et al., 2007; Jucker, 2010; Drummond and Wisniewski, 2017; Esquerda-Canals et al., 2017). These models, however, remain crucial for testing experimental interventions like gamma-tACS across various pathological aspects before clinical use. APP and APP/PS1 mice are commonly used amyloidosis models due to their rapid Aβ plaque accumulation, though they lack significant neurodegeneration and tau pathology (Radde et al., 2006; Serneels et al., 2020). Similarly, 5XFAD model, which exhibits rapid amyloid deposition and memory deficits, is suitable for studying neuroinflammation but lacks tauopathy (Oakley et al., 2006; Jawhar et al., 2012; Richard et al., 2015). For tauopathy, the A152T tau model allows the study of both aggregate (Decker et al., 2016; Sydow et al., 2016) and soluble (Maeda et al., 2016) tau effects on synaptic activity. The APOE knock-in model offers insights into the role of APOE isoforms in Aβ deposition and neuroinflammatory responses, relevant to late-onset AD (Huynh et al., 2019). The 3xTg-AD model combines mutations in APP, PS1, and tau, exhibiting both amyloidosis and tauopathy, though it does not fully replicate human AD neurodegeneration or microglial/inflammatory responses (Oddo et al., 2003). Although no model fully replicates human AD, diverse models enable researchers to study specific pathological mechanisms, being essential for evaluating experimental treatments like gamma-tACS. In recent studies, Wu et al. (2022, 2023) showed how gamma-tACS in the APP/PS1 model improved cognitive function, modulated microglial activity, and reduced amyloid burden, indicating its potential to affect neuroinflammation and connectivity. Similarly, Jeong et al. (2021) found that 40 Hz gamma-tACS enhanced hippocampal LTP in 5XFAD mice, improving memory and plasticity without impacting markers like BDNF or CREB. These findings suggest gamma-tACS as a promising method to strengthen synaptic connectivity and memory, potentially addressing cognitive deficits in AD models.

### Clinical studies of tACS in AD

Numerous clinical studies have explored multisession gamma-tACS protocols in AD patients, showing potential cognitive and neural benefits consistent with preclinical findings that gamma oscillations may reduce Aβ deposits and enhance memory pathways through neural entrainment and neuroplasticity. Sprugnoli et al. (2021), Altomare et al. (2023) and Cappon et al. (2023) demonstrated cognitive improvements with 40 Hz-tACS over multi-week interventions. Sprugnoli et al. (2021) applied 40 Hz-tACS to temporal regions, increasing hippocampal perfusion and episodic memory. Altomare et al. (2023) targeted the precuneus with home-based tACS over 16 weeks, using EEG and Positron Emission Tomography (PET) to show connectivity-linked cognitive gains, while Cappon et al. (2023) observed episodic memory improvements with angular gyrus stimulation over 14 weeks. Furthermore, Liu et al. (2023) combined sound with 40 Hz-tACS, finding enhanced memory and connectivity, suggesting that multisensory stimulation could amplify gamma-tACS effects. Additionally, Benussi et al. (2021) evaluated the impact of gamma-tACS in early stages of AD, finding significant cognitive improvements. Naro et al. (2016) suggested that increases in gamma-band oscillations could indicate progression risk, as patients displaying them together with cognitive improvements were less likely to progress to AD over a two-year period. These studies underscore gamma-tACS’s potential as a non-invasive intervention to enhance episodic memory and connectivity in AD. Consistent cognitive improvements across extended gamma-tACS protocols support its utility for cognitive network modulation, while long-term studies are needed to confirm the durability of these effects.

### Animal models of PD and preclinical insights

PD research relies on animal models to replicate key pathological features like dopaminergic neurodegeneration, alpha-synuclein aggregation, and neuroinflammation (Hunt et al., 2022). Common models include the toxin-induced MPTP and 6-OHDA, selectively damaging nigrostriatal dopaminergic neurons to mimic PD motor symptoms, though they lack *α*-synuclein pathology and disease progression (Ungerstedt, 1968; Ovadia et al., 1995). To model α-synuclein aggregation, transgenic and viral vector models allow for human α-synuclein overexpression, leading to progressive dopaminergic deficits and neuroinflammatory responses, as demonstrated in the viral vector model by Decressac et al. (2012). Genetic models, targeting LRRK2 and GBA1, offer insights into PD risk factors; LRRK2 models affect mitochondrial function and autophagy (Tsika et al., 2014), while GBA1 models demonstrate glucocerebrosidase deficiency and moderate α-synuclein buildup, aiding the study of lysosomal dysfunction in PD pathology and therapies (Polinski et al., 2021). Although limited, these diverse models provide essential insights into dopaminergic loss, protein aggregation, and neuroinflammation, advancing PD pathology understanding and therapeutic research. Preclinical studies on tACS in PD are sparse. Although tACS has shown benefits for motor and cognitive symptoms in clinical PD studies, little research explores its mechanisms and therapeutic potential in PD models. This gap highlights the need for foundational studies to determine how frequency-specific tACS could modulate dopaminergic circuits, plasticity, and neuroinflammation. A starting point is Lee et al. (2022), who examined tACS effects in an MPTP model, finding that beta-tACS improved motor performance, likely due to its resonance with basal ganglia oscillations, while gamma-tACS did not show similar benefits. Further research is needed to identify optimal tACS parameters in PD models to inform future clinical applications.

### Clinical studies of tACS in PD patients

Although preclinical studies on tACS in PD models are limited, substantial clinical research has examined tACS at various frequencies in PD patients, revealing its therapeutic potential, particularly for motor and cognitive symptoms. Studies highlight the effects of gamma-tACS, especially at 70 Hz, in enhancing cortical plasticity and motor function. Guerra et al. (2020) demonstrated that 70 Hz-tACS with intermittent theta burst stimulation (iTBS) over the motor cortex (M1) restored LTP-like plasticity in PD patients, particularly those with shorter disease duration, suggesting gamma oscillations may compensate for plasticity deficits. Furthermore, Guerra et al. (2023a) found that gamma-tACS combined with iTBS increased motor-evoked potential (MEP) facilitation and GABA-Aergic function in M1, with similar effects regardless of patients’ dopaminergic state, indicating that gamma-tACS may work synergistically with dopaminergic therapies. In studies on motor impairments, Guerra et al. (2022) found that gamma-tACS at 70 Hz increased movement amplitude, while beta-tACS (20 Hz) improved movement speed, suggesting that both oscillations may address different aspects of motor control. Guerra et al. (2023b) also showed that beta-tACS combined with repetitive transcranial magnetic stimulation (rTMS) restored short-term plasticity (STP) in PD patients, reducing bradykinesia severity by modulating GABA-A circuits, independently of medication. These clinical findings emphasize tACS’s potential for targeting PD motor symptoms with frequency-specific effects, from movement amplitude to coordination. However, discrepancies between human and preclinical results underscore the need for more animal research to clarify tACS mechanisms across species, refining protocols for optimal translational applications in PD treatment.

## Preclinical tACS research: addressing complexities for clinical translation

Although various neurological conditions are associated with alterations in gamma oscillations, it is critical to acknowledge that these alterations are driven by multiple factors. This complexity suggests that attributing them to a single cause oversimplifies the nuanced nature of neural function (Buzsáki and Wang, 2012; Fries, 2015). It is also important to recognize that gamma stimulation can induce multiple effects that extend beyond merely imposing the gamma frequency on the affected neuronal populations. Thus, in exploring tACS, it is essential to address the intricate nature of gamma oscillations and the challenge of identifying a singular causative mechanism for their anomalies (Uhlhaas and Singer, 2010). This multifactorial nature underscores the necessity for a comprehensive understanding of the effects of tACS across different biological levels—from molecular to systemic—before its application can be effectively translated to clinical settings. Such an understanding involves a meticulous exploration of how tACS influences neuronal and network behavior in animal models, which may pave the way for targeted interventions in human subjects (Herrmann et al., 2013; Polanía et al., 2018). By expanding our grasp on these mechanisms, we enhance the foundational knowledge in preclinical tACS research, which is crucial for the successful design of tACS protocols tailored to address specific neurological deficits. This approach not only maximizes therapeutic outcomes but also minimizes assumptions about the direct translatability of frequency-specific interventions, promoting a more nuanced and effective application of tACS in clinical practice (Reato et al., 2010; Vossen et al., 2015). Animal models provide an essential controlled environment to investigate how anatomical and physiological factors influence the outcomes of tACS (Huang et al., 2017; Vöröslakos et al., 2018). These models are crucial for optimizing electrode placement and stimulation parameters to accommodate structural differences (Huang et al., 2021), and they facilitate high-resolution intracranial recordings that help identify ideal stimulation frequencies and intensities (Reato et al., 2010; Farahani et al., 2024). However, notable differences in factors such as skull density and experimental procedures like open-head stimulation in these models often amplify the observed effects of tACS (Jackson et al., 2016). These discrepancies highlight the challenges in directly extrapolating results to humans, underscoring the need for rigorous translational research to adapt findings from animal studies to clinical applications effectively (Sánchez-León et al., 2018).

## Conclusion

Gamma-tACS holds promise as a neuromodulation tool for neurodegenerative diseases, especially AD. Preclinical studies indicate its ability to modulate neural oscillations, enhance cognitive function, and provide neuroprotective effects, like reducing Aβ deposits and influencing microglial activity. Clinical trials in AD patients suggest that gamma-tACS can improve episodic memory and connectivity, complementing similar studies with sensory stimulation at gamma frequencies and supporting its role as a non-invasive treatment for cognitive decline. However, more preclinical studies in PD are needed to explore its impact on dopaminergic circuits, motor symptoms, and neuroinflammation. Insights from animal models could refine personalized protocols, optimizing stimulation parameters for individual differences. Bridging preclinical and clinical insights, gamma-tACS could enhance cognitive resilience and slow disease progression across neurodegenerative conditions.
